# Current Knowledge in Thyroid Cancer—From Bench to Bedside

**DOI:** 10.3390/ijms18071529

**Published:** 2017-07-15

**Authors:** Daniela Grimm

**Affiliations:** 1 Department of Biomedicine, Pharmacology, Aarhus University, Wilhelm Meyers Allé 4, 8000 Aarhus C, Denmark; dgg@biomed.au.dk or daniela.grimm@med.ovgu.de; Tel.: +45-8716-7693; Fax: +45-8612-8804; 2 University Clinic for Plastic, Aesthetic and Hand Surgery, Otto von Guericke University, Leipziger Str. 44, 39120 Magdeburg, Germany

Thyroid cancer is the most common malignant endocrine tumour. Differentiated thyroid cancer (DTC) is a rare disease with increased incidence. In 2014, 62,980 (15,190 for males and 47,790 for women) cases of DTC were estimated in the US, compared to 2009 with 31,200 new cases [1]. In the US, estimated new cases for 2017 are 14,400 for males and 42,470 for females [2]. Thyroid cancer incidence rates are three-fold higher in women than in men (21 vs. 7 per 100,000 population), despite equivalent death rates (0.5 per 100,000 population) [2]. Worldwide data for thyroid cancer are available from GLOBOCAN 2012. The incidence of thyroid cancer in 2012 was estimated to be 298,102 new cases worldwide, representing 2.1% of all cancers [3].

Thyroid cancer types are classified according to their histological characteristics. DTC types comprise papillary thyroid cancer (PTC) and follicular thyroid cancer (FTC). DTC represents the majority of all thyroid carcinoma types with a generally positive prognosis [4]. The next group is anaplastic thyroid cancer (ATC), representing approximately 2% of all thyroid cancers. ATC is different from DTC, in that the cells mainly consist of undifferentiated cells with a very low or no similarity to normal thyroid tissue [5]. The one-year mortality rate is 90% [6]. Poorly differentiated thyroid cancer (PDTC) is similar to ATC; it is less common and has a poor prognosis [7]. Another thyroid cancer type is medullary thyroid cancer (MTC), deriving from neuroendocrine C-cells. The five-year survival rate is approximately 86% for MTC patients [8].

In recent years, studies in the field of thyroid cancer research have been performed in order to improve diagnosis with molecular biological techniques, to extend treatment strategies, to identify and verify thyroid specific biomarkers, as well as to identify cancer-specific changes in gene expression patterns and alterations of the protein content. Furthermore, new drugs, small molecules, and antibodies have been developed and tested in vitro and in vivo [9,10]. In clinical trials, the ratio between therapeutic and adverse effects was investigated [10]. Tyrosine kinase inhibitors (TKI) have become a new therapeutic option in patients with radioactive iodine (RAI)-refractory progressive DTC and in medullary thyroid cancer. In the last few years, new substances for targeted systemic therapy have been approved after their efficacy was demonstrated in Phase III trials. Most of them show a moderate response. However, adverse effects are common and have to be managed, such as hypertension, fatigue, diarrhoea, and others.

In this Special Issue, a total of 16 excellent papers consisting of nine original research studies, six reviews, and one communication have been published, as detailed in Table 1.

Several manuscripts focusing on the diagnosis [11,12,13,14,15], characterisation [16,17,18,19], and treatment [20,21,22] of thyroid cancer, as well as on alterations of thyroid cancer cells by altered gravity [23,24] or radiation damage of the thyroid gland [25], including genetics, proteomics, molecular, and cell biology, are published in this Special Issue. In addition, an original publication reports that the polymorphism loci of the tumour necrosis factor superfamily member 4 (*TNFSF4*) gene is related to early-onset autoimmune thyroid diseases [26].

This Special Issue also covered studies on patients, providing novel mechanistic insights into the thyroid cancer pathogenesis and new aspects that may impact diagnosis or clinical therapy. Recent study results in order to review the current status of new therapy options in thyroid cancer were published [20,21].

Fine needle aspiration cytology (FNAC) is the gold standard for the investigation of thyroid nodules, often occurring in regions with iodine deficiency, but also in countries with iodine prophylaxis. Cantara et al. [11] provided a review on thyroid molecular testing to determine the nature of thyroid nodules and showed data on mutational FNAC material analysis and circulating micro-ribonucleic acid (miRNA) expression. Available commercial tests to identify cancer have to include *BRAF* and *RAS* point mutations, as well as *RET/PTC*, *NTRK*, and *PAX8/PPARg* rearrangements. The identification of new biomarkers such as miRNA, circulating cancer cells, and proteomic profiling are proposed to discriminate benign from malignant thyroid nodules, but future research is still necessary.

Ko et al. [12] showed that the diagnostic limitation of FNA on indeterminate nodules might be partially overcome by a molecular analysis before surgery. *RET/PTC1* rearrangement by RT-PCR analysis on air-dried FNA specimens could be technically applicable. A significant proportion (38.2%) of the *BRAF* and *RAS* wild type PTCs harbour a *RET/PTC1* rearrangement. This preoperative molecular analysis is a helpful diagnostic examination of FNA specimens to characterise indeterminate thyroid nodules [12].

Biomarkers for cancer are currently a hot topic. One such example is the cell-free DNA (cfDNA) quantity and quality in plasma. The study by Salvianti et al. [13] demonstrated that the cfDNA integrity index 180/67 is a suitable parameter for monitoring cfDNA fragmentation in thyroid cancer patients. In fact, cfDNA quantity and integrity were higher in patients affected by modular thyroid diseases than in healthy controls. In addition, these markers might be helpful in the diagnosis of thyroid nodules [13].

The next study focused on mRNA markers for differentiating between malignant and benign follicular tumours [14]. Wojtas et al. [14] analysed gene expression microarray data and performed a meta-analysis of 14 studies employing high throughput methods. They showed that several genes are differentially expressed in follicular thyroid carcinoma (FTC) and follicular thyroid adenoma (FTA). They created a classifier that distinguished between FTC and FTA based on the gene expression of *CPQ*, *PLVAP*, *TFF3*, and *ACVRL1*. The authors concluded that there is a plausible limit of gene classifier accuracy of 80%, when follicular neoplasms are diagnosed based on formalin-fixed specimen post-surgery [14].

Schob et al. [15] showed that histogram analyses of diffusion weighted imaging (DWI) at 3T are useful for the prediction of tumour progression in the contest of lymphatic spread, proliferation, and cellularity in thyroid cancer. The authors used DWI histogram analysis of whole tumour apparent diffusion coefficient (ADC) maps. This method can provide information in tumour biology in thyroid cancer. Significant correlations between ADC parameters were identified with p53, ki-67, and cell count [15].

The next group of publications mainly focused on cancer characterisation [16,17,18,19]. The study of Ruggeri et al. [16] described the human epidermal growth factor receptor 2 (HER2) status in sporadic thyroid carcinomas of follicular cell origin. The authors investigated 45 FTC and 45 PTC surgical specimens. HER2 overexpression was detected in 44% of the FTC and 18% of the PTC samples. Five of six patients with a HER2-positive cancer developed a metastatic disease during a median nine-year follow-up [16].

Caria et al. [17] investigated the genetic heterogeneity of HER2 amplification and telomere shortening in PTC. The association of HER2 amplification with *BRAF^V600E^* mutation and telomere shortening and prevalence of multifocal tumours may characterise a subgroup of familial PTC [17].

Su et al. [18] published a comprehensive characterisation of the mitochondrial genome of 66 PTC, 16 normal thyroid tissues, and 376 blood samples. This data demonstrate the significance of the mitochondrial genome in the pathogenesis (mtDNA mutations, single-nucleotide polymorphisms (mtSNPs)) and progression of PTC. These findings help to reveal the molecular mechanism underlying PTC and may offer new biomarkers and possible targets for new treatment options [18].

Steiner et al. [19] focused on the expression of tenascin C, E-cadherin, EGFR, and thyroid transcription factor 1 (TTF-1) in medullary thyroid carcinomas (MTC). Tenascin C found in all MTC correlated with proliferation, mainly in RET-mutated tumours. The EGFR expression was low and EGFR-positive tumours do not exhibit a higher proliferation [19].

Schmidbauer et al. [20] reviewed the state-of-the-art DTC treatment strategies. The authors gave an overview of the TNM (**T**umour size, involvement of lymph **N**odes, **M**etastasis) classification of malignant tumours for thyroid cancer types, summarised the diagnostic approaches to thyroid nodules, and reviewed the current therapy of DTC [20]. In addition, the authors studied the application of tyrosine kinase inhibitors (TKI) in poorly differentiated thyroid cancer without iodine metabolism. Unfortunately, these drugs have adverse effects. The adverse effect of hypertension in the treatment of thyroid cancer with multi-kinase inhibitors (MKI), was reviewed by Ancker et al. [21]. Clinical trials have shown that a large number of thyroid cancer patients treated with MKI were affected by hypertension [21]. Different antihypertensive drugs used to manage the TKI-induced hypertension were discussed [21].

Manzella et al. [22] provided an update on the current knowledge of the impact of p53 family proteins in thyroid cancer. In addition, their possible role as therapeutic targets for the treatment of undifferentiated cancer is discussed. p53 family members are involved in the development of multiple thyroid cancer types. Therefore, therapeutic molecules targeting these proteins are of high interest and should be available for therapy soon.

Gravitation force has an enormous influence on the human body and on single organ functions. Altered gravity conditions can seriously influence the health of crewmembers after a long-term spaceflight and induce a variety of physiological changes [27,28,29]. Albi et al. [23] reviewed the impact of simulated and real microgravity and hypergravity on normal thyrocytes and on thyroid cancer cells. The identification of new mechanisms of the thyroid injury may be important for the development of countermeasures for crewmembers, as well as for those on Earth. The altered gene expression and changed protein content evaluated by proteomics studies on thyroid cancer cells may be helpful to find new target proteins of interest. Bauer et al. [24] performed a proteome analysis of human FTC cells exposed to a random positioning machine, a device designed to simulate a microgravity-like environment on Earth. The FTC cells grew as an adherent monolayer and as three-dimensional aggregates on this device. In this communication, the authors showed that the proteins paxillin (PXN), vinculin (VCL), and focal adhesion kinase 1 (PTK2/FAK1) may be positioned within the focal adhesion complex in a way that favours cell detachment from the cell culture flask bottom and mutual attachment [24].

In a second review, Albi et al. [25] reported on radiation-induced thyroid injury. Radiation induces apoptosis, cell cycle arrest, DNA repair, and cancer in the thyroid gland. The authors provided an overview of radiation effects on the thyroid gland and on cancer development. Various molecular targets are identified by which radiation damages the thyroid gland. In addition, the paper describes the regulation of lipids in response to radiation-induced damage and their involvement in thyroid cancer.

Finally, the research article by Song et al. [26] showed that *TNFSF4* gene variations are related to early-onset autoimmune thyroid diseases and hypothyroidism of Hashimoto’s thyroiditis in China. In addition, these results supported the importance of T cells in the pathology of autoimmune thyroid diseases [26].

Overall, the 16 important contributions published in this Special Issue illustrate the advances in the field of thyroid cancer research as well as autoimmune thyroid disease.

I would like to thank all the authors who contributed to this Special Issue, and I remain hopeful that increasing knowledge of how to diagnose, treat, and prevent thyroid cancer as well as finding new proteins which may serve as targets for new drug design will help to reduce the incidence and mortality of this cancer type.

## Figures and Tables

**Table 1:** Contributions to the Special Issue “Current Knowledge in Thyroid Cancer—From Bench to Bedside”.

Authors	Title	Topics	Type
Cantara et al. [11]	Molecular Signature of Indeterminate Thyroid Lesions: Current Methods to Improve Fine Needle Aspiration Cytology (FNAC) Diagnosis	Diagnosis	Review
Ko et al. [12]	Diagnostic Limitation of Fine-Needle Aspiration (FNA) on Indeterminate Thyroid Nodules Can Be Partially Overcome by Preoperative Molecular Analysis: Assessment of *RET/PTC1* Rearrangement in *BRAF* and *RAS* Wild-Type Routine Air-Dried FNA Specimens	Diagnosis	Original research
Salvianti et al. [13]	Integrity and Quantity of Total Cell-Free DNA in the Diagnosis of Thyroid Cancer: Correlation with Cytological Classification	Diagnosis	Original research
Wojtas et al. [14]	Gene Expression (mRNA) Markers for Differentiating between Malignant and Benign Follicular Thyroid Tumours	Diagnosis	Original research
Schob et al. [15]	Histogram Analysis of Diffusion Weighted Imaging at 3T is Useful for Prediction of Lymphatic Metastatic Spread, Proliferative Activity, and Cellularity in Thyroid Cancer	Diagnosis	Original research
Ruggeri et al. [16]	HER2 Analysis in Sporadic Thyroid Cancer of Follicular Cell Origin	Characterisation	Original research
Caria et al. [17]	Genetic Heterogeneity of *HER2* Amplification and Telomere Shortening in Papillary Thyroid Carcinoma	Characterisation	Original research
Su et al. [18]	Comprehensive Characterisation of Mitochondrial Genome in Papillary Thyroid Cancer	Characterisation	Original research
Steiner et al. [19]	Expression of Tenascin C, EGFR, E-Cadherin, and TTF-1 in Medullary Thyroid Carcinoma and the Correlation with *RET* Mutation Status	Characterisation	Original research
Schmidbauer et al. [20]	Differentiated Thyroid Cancer Treatment: State of the Art	Therapy	Review
Ancker et al. [21]	The Adverse Effect of Hypertension in the Treatment of Thyroid Cancer with Multi-Kinase Inhibitors	Therapy	Review
Manzella et al. [22]	New Insights in Thyroid Cancer and p53 Family Proteins	Therapy	Review
Albi et al. [23]	Impact of Gravity on Thyroid Cells	Alterations	Review
Bauer et al. [24]	Proteome Analysis of Human Follicular Thyroid Cancer Cells Exposed to the Random Positioning Machine	Proteomics	Communication
Albi et al. [25]	Radiation and Thyroid Cancer	Radiation Damage	Review
Song et al. [26]	*TNFSF4* Gene Variations Are Related to Early-Onset Autoimmune Thyroid Diseases and Hypothyroidism of Hashimoto’s Thyroiditis	Benign Thyroid disease	Original research

## Abbreviations

*BRAF*	B-Raf (rapidly accelerated fibrosarcoma) proto-oncogene
cfDNA	Cell-free deoxyribonucleic acid
DTC	Differentiated thyroid cancer
EGFR	Epidermal growth factor receptor
FNA	Fine needle aspiration
FNAC	Fine needle aspiration cytology
FTC	Follicular thyroid cancer
HER2	Human epidermal growth factor receptor 2
MKI	Multi-kinase inhibitors
MTC	Medullary thyroid cancer
miRNA	Micro-ribonucleic acid
PTC	Papillary thyroid carcinoma(s)
PTK2/FAK1	Focal adhesion kinase 1
PXN	Paxillin
RAS	Rat sarcoma proto-oncogene
TKI	Tyrosine-kinase inhibitors
*TNFSF4*	Tumour necrosis factor superfamily member 4
TNM	TNM Classification of Malignant Tumours
TTF1	Thyroid transcription factor 1
VCL	Vinculin
